# Sugar Shock: Probing *Streptococcus pyogenes* Metabolism Through Bioluminescence Imaging

**DOI:** 10.3389/fmicb.2022.864014

**Published:** 2022-06-02

**Authors:** Richard W. Davis, Charlotte G. Muse, Heather Eggleston, Micaila Hill, Peter Panizzi

**Affiliations:** Department of Drug Discovery and Development, Auburn University, Auburn, AL, United States

**Keywords:** bioluminescence, biosensor, pathogens, physiology, *Streptococcus pyogenes*, molecular imaging

## Abstract

*Streptococcus pyogenes* (*S. pyogenes*) can thrive in its host during an infection, and, as a result, it must be able to respond to external stimuli and available carbon sources. The preclinical use of engineered pathogens capable of constitutive light production may provide real-time information on microbial-specific metabolic processes. In this study, we mapped the central metabolism of a *luxABCDE*-modified *S. pyogenes* Xen20 (*Strep*. Xen20) to its *de novo* synthesis of luciferase substrates as assessed by the rate of light production in response to different environmental triggers. Previous characterization predicted that the *lux* operon was under the myo-inositol *iolE* promotor. In this study, we revealed that supplementation with myo-inositol generated increased *Strep.* Xen20 luminescence. Surprisingly, when supplemented with infection-relevant carbon sources, such as glucose or glycine, light production was diminished. This was presumably due to the scavenging of pyruvate by *L*-lactate dehydrogenase (LDH). Inhibition of LDH by its inhibitor, oxamate, partially restored luminescent signal in the presence of glucose, presumably by allowing the resulting pyruvate to proceed to acetyl-coenzyme A (CoA). This phenomenon appeared specific to the lactic acid bacterial metabolism as glucose or glycine did not reduce signal in an analogous *luxABCDE*-modified Gram-positive pathogen, *Staph*. Xen29. The *Strep.* Xen20 cells produced light in a concentration-dependent manner, inversely related to the amount of glucose present. Taken together, our measures of microbial response could provide new information regarding the responsiveness of *S. pyogenes* metabolism to acute changes in its local environments and cellular health.

## Introduction

*Streptococcus pyogenes* (*S. pyogenes*) infections often manifest as necrotizing fasciitis or cellulitis (Carapetis et al., 2005). Surveillance Report for 2019 reported 25,050 new cases of *S. pyogenes* infections for every 34.6 million individuals. The incidence of cellulitis or necrotizing fasciitis over the same period was 44.7 and 4.5%, respectively. The total rate for 2019 showed 7.6 per 100,000 cases, and 9% of those resulted in death (Centers for Disease Control and Prevention, 2019).

An intriguing preclinical method for real-time *in vivo* tracking of these harmful pathogens during an infection is bioluminescence. Although the use of engineered light-producing microbes has a limited diagnostic value, the benefit of these types of bacteria for preclinical experimentation is often underappreciated. For example, deposition of bacterial-targeted dyes *in vivo* during preclinical testing can be readily verified by co-localization with the light signal from one of these engineered microbes. Typically, such studies require that the parental strain be transformed with plasmids containing either the *Photorhabdus luminescens lux* operon cassette (*luxABCDE*) or the firefly luciferase (*ffluc*) enzyme. One significant limitation to the use of these pathogens is the gap in our understanding regarding changes in the light production by these microbes. The *luxABCDE* cassette often is placed on a transposable element, so expression is dependent on its random insertion into the genome of the parent bacterium (Chu et al., 2009). As such, light production can be linked to cellular processes that are inherent to the microbe and report on the microbes’ response to its local environments. Previously, we studied a *luxABCDE*-modified *S. pyogenes* Xen20 (*Strep*. Xen20) to track the spread of cutaneous infection in wild-type mice (Davis et al., 2015). By *ex vivo* colony forming units’ (CFUs) determination and Gram-staining histology, we noted live *Streptococcus* present in the distal organs of the infected animals. Our findings did not support the non-invasive bioluminescent imaging (BLI) results, suggesting either the signal was below the limit of detection or there was a breakdown of the light production *in vivo*. We found that light production by *Strep.* Xen20 decreased with the increasing *D*-glucose concentration, thereby, essentially serving as a glucose biosensor within the animal. Recently, Mimee et al. (2018) reported on a similar light-producing *Escherichia coli* (*E. coli*) used in an ingestible bacterial-electronic system that detects the presence of heme in a model of gastric bleeding. Such microbial systems or devices could serve as biosensors in venues ranging from clinical to industrial.

Light production by the emitting microbes follows the conversion of activated fatty acyl compounds to fatty aldehydes *via* the *luxCDE* system (Meighen, 1993). These fatty aldehydes drive the *luxAB* complexes, which use molecular oxygen and reduced flavin mononucleotide (FMNH_2_) to produce fatty acid, water, oxidized flavin mononucleotide, and light. Therefore, an essential requirement of bioluminescence is the availability of critical reactants, such as non-anoic acid, FMNH_2_, and adenosine triphosphate (ATP) (Meighen, 1991). In this study, we showed a differential light production based on catabolite repression and gluconeogenesis for streptococcal and staphylococcal strains of similar *lux*-cassette design. Furthermore, we challenged these microbes with different stimuli (i.e., myo-inositol, oxamate, and carbon sources). We monitored their relative light production to dissect processes that would promote or repress the light production pathway in these strains. Given our results, it may be possible to use these light-producing pathogens as biosensors for the rapid assessment of physiologic processes and the local microbial environment.

## Results

### *D*-Glucose Inhibits Bioluminescence From *Strep.* Xen20

**FIGURE 1:**
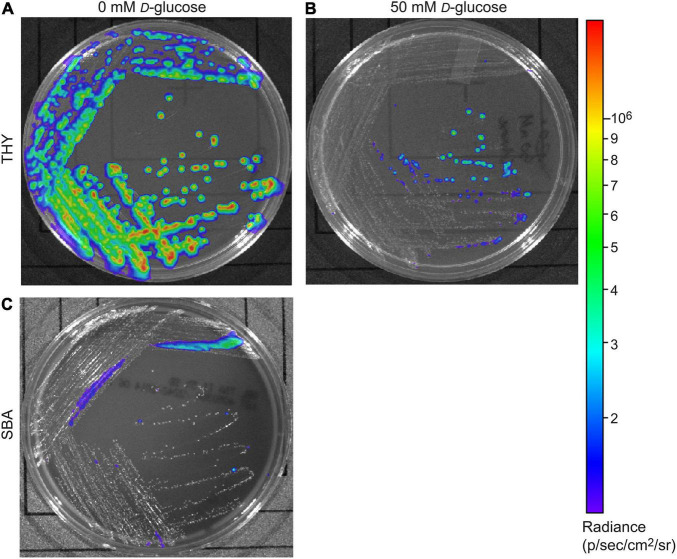
Glucose effect on bioluminescent production of *S. pyogenes* Xen20. *Strep.* Xen20 was plated on Todd-Hewitt with yeast extract (THY) without **(A)** or with the addition of excess D-glucose **(B)** or on sheep blood agar (SBA) and incubated for 20 h at 37°C **(C)**.

Our previous results indicated that the luminescence of *Strep.* Xen20 did not accurately reflect bacterial load *in vivo* and suggested this may be due to a *D*-glucose-mediated inhibitory effect (Davis et al., 2015). To confirm this finding on a static medium, we plated *Strep*. Xen20 on Todd-Hewitt with yeast extract (THY) plates supplemented with 0 or 50 mM *D*-glucose. For this experiment, cells were grown overnight and imaged for luminescence (Figure 1). Colonies with *D*-glucose supplementation showed decreased luminescence. Interestingly, the presence of sheep blood (SB) also significantly reduced the bioluminescence produced by *Strep.* Xen20. Similar CFUs were also observed by colony counting.

### The *lux* Operon in *Strep.* Xen20 Is Regulated by Inositol and Glucose Levels

**FIGURE 2:**
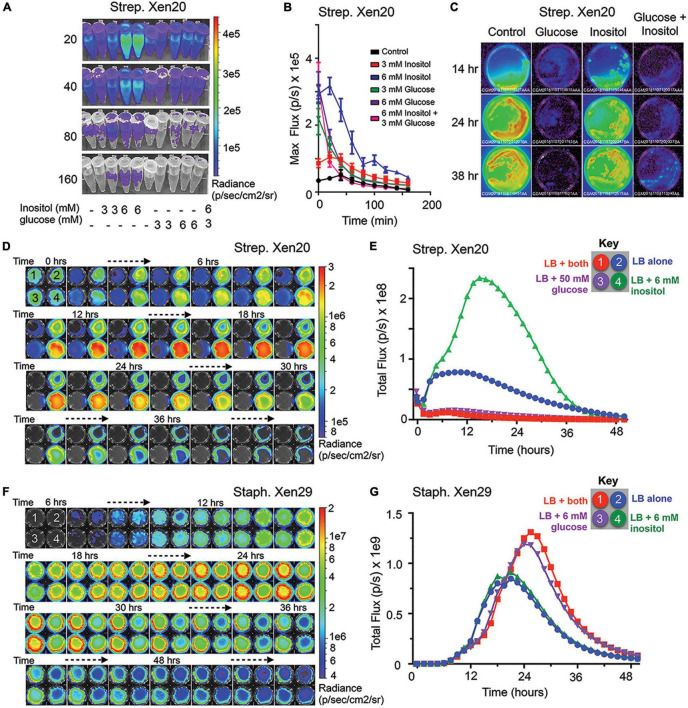
Myo-inositol regulates luminescence of *S. pyogenes* Xen20. **(A)** Representative time course of *Strep.* Xen20 cells. Samples were grown to the stationary phase and then diluted in fresh phosphate-buffered saline (PBS) supplemented with one of either 3 or 6 mM myo-inositol or 3 or 6 mM *D*-glucose. **(B)** Quantification of luminescent signals from samples shown in panel **(A)** shown as the maximum rate of photons generated (max flux). **(C)** Bioluminescent imaging (BLI) of 65 mm Luria broth (LB) plates containing no additive (control), 50 mM glucose, 6 mM myo-inositol, or both taken at 14, 24, and 38 h after incubating at 37°C. **(D)** *Strep.* Xen20 growth on 6-well, flat-bottomed culture plates coated with LB agar containing either both myo-inositol and glucose (1, indicated in red), no additive (2, blue), 50mM glucose (3, purple), or 6 mM myo-Inositol (4, green) and imaged over 48 h in the IVIS. **(E)** BLI quantification of the plates from panel **(D)** as the total rate of photons generates (Total flux). **(F)** *Staph*. Xen29 incubated on 6-well, flat-bottomed culture plates coated with LB agar containing either no additive (control), 6 mM glucose, 6 mM myo-Inositol, or both and imaged over 60 h in the IVIS. **(G)** BLI quantification of the plates from panel **(D)** as the total rate of photons generates (Total flux).

*Strep.* Xen20 has the *luxABCDE* cassette inserted in the *iol* operon used for inositol catabolism. More specifically, the gene is under the *iolE* promoter (Park et al., 2003), which encodes 2-keto-myo-inositol dehydratase (Yoshida et al., 2004). The IolR repressor controls the entire *iol* operon (Yoshida et al., 1999). Under high inositol conditions, the repressor is liberated, and genes are expressed. As such, we tested the contribution of inositol to bioluminescence expression in *Strep*. Xen20. The results (Figure 2A) indicate increased light from bacterial cells grown in the presence of myo-inositol. In contrast, co-supplementation of *Strep.* Xen20, with both inositol and a low concentration of glucose (3 mM), overcame this inositol-dependent enhancement. All BLI signal returned to baseline in less than 3 h (Figure 2B). To evaluate this inositol benefit in actively growing cells, equal portions of *Strep*. Xen20 were spread on Luria broth (LB) plates in the absence or the presence of 6 mM myo-inositol, of 50 mM *D*-glucose, or both additives (Figure 2C). Results paralleled Figure 1 and the tube tests in Figures 2A,B. BLI from bacterial plates at 14, 24, and 38 h in the presence of *D*-glucose plates showed near-complete loss of light production, and this was only slightly counteracted by the addition of myo-inositol at the 38 h time point (Figure 2C).

To support cross-conditional comparisons and to coordinate timing, we imaged the four distinct conditions simultaneously over 2 days, i.e., the *Strep*. Xen20 kinetic profile showed a definite 2-reaction trace with the development of a new inositol peak at 16–17 h that was almost, approximately, fourfold greater than the amount of light emitted by the LB control (Figures 2D,E). Despite similar bacterial growth over the time course, the BLI signal for both the 50 mM *D*-glucose- and 6 mM myo-inositol-supplemented conditions showed markedly lower light production. Conversely, *Staph.* Xen29 showed no change in light production with 6 mM inositol supplementation alone and an approximately twofold increase when grown in the presence of *D*-glucose that was inositol-independent (Figures 2F,G). These results agree with previous research that showed the expression of green fluorescent protein (GFP), placed under the *iolE* promoter, was decreased in glucose-rich conditions under the *iolE* promoter in *Salmonella enterica* serovar *Typhimurium* strain 14028 (Kroger et al., 2011).

### Substrate Sources for Bioluminescence of *Streptococcus pyogenes*

**FIGURE 3:**
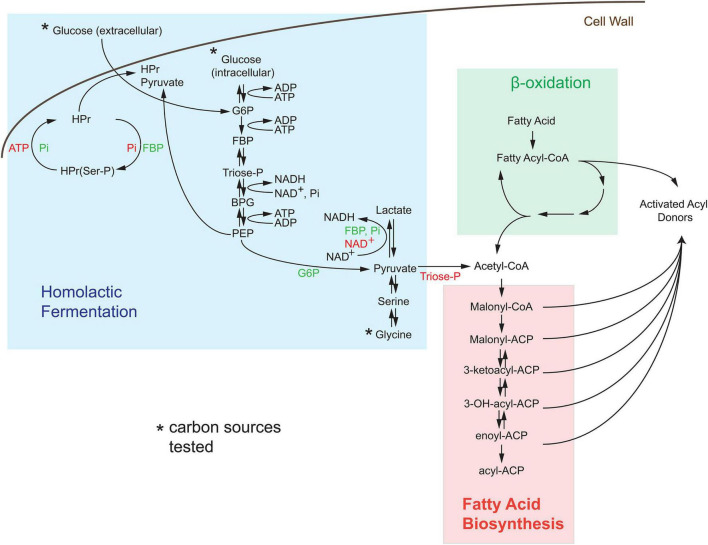
*De novo* synthesis of acyl donors *via* central carbon metabolism in *Streptococcus*. Activated acyl donors, the substrates for the *luxABCDE* machinery, are made by the breakdown of fatty acids through β-oxidation (denoted as green region) or by the synthesis of an activated carrier protein (ACP)-containing metabolites *via* fatty acid biosynthesis (denoted as salmon region). Since *S. pyogenes* lacks the β-oxidation pathway, all substrates for light production in *Strep*. Xen20 are created by acetyl-coenzyme A (CoA)-dependent fatty acid biosynthesis generated by homolactic fermentation (denoted as blue box). The asterisk indicates the sole carbon sources tested. Allosteric activators, such as inorganic phosphate (Pi), fructose bisphosphate (FBP), and glucose-6-phosphate (G6P) are shown in green text, and allosteric inhibitors, including adenosine triphosphate (ATP), nicotinamide adenine dinucleotide (NAD +), and triose phosphate (triose-P), are shown in red text. Abbreviations used in here is as follows: histidine-containing carrier protein (HPr), phosphorylated HPr [HPr(Ser-P)], phosphoenolpyruvate (PEP), and 1,3-bisphosphoglyceric acid (BPG).

Since inositol cannot function as a carbon source in *S. pyogenes* M49 strains [based on the Kypto Encyclopedia of Genes and Genomes (KEGG) genomic data], we investigated the agonistic or antagonistic effects of central metabolites on the production of light. As previously stated, bacterial bioluminescence is dependent on the production of substrates by the bacterial cell (Meighen, 1993); therefore, we investigated the link of bioluminescence to central metabolism in *S. pyogenes* (see pathway scheme in Figure 3). Bioluminescence is entirely dependent on the availability of activated acyl donors, which are produced *de novo* by the bacterial cell and serve as the substrate for the *lux* operon to create fatty aldehydes. These substrates are then converted to fatty acids in the presence of FMNH_2_ and oxygen, thereby releasing light. Activated acyl donors are synthesized in one of two ways, first, coenzyme A (CoA)-containing acyl compounds can be created *via* the breakdown of fatty acids by β-oxidation (green box, Figure 3); second, CoA-containing fatty acid building blocks can be created *via* the fatty acid biosynthesis pathway (salmon box, Figure 3). The second pathway is dependent upon the creation of acetyl-CoA by the glycolysis/lactic acid fermentation pathway (blue box, Figure 3).

Although the KEGG pathways are not available for *Strep.* Xen20, a pathway exists for the closely related M49 serotype ancestor NZ131^1^ (Mcshan et al., 2008). The analysis of this pathway revealed important allosteric and feedback mechanisms for bioluminescence production. First, M49 strains lack the β-oxidation path beyond the creation of hexadecanoyl-CoA from hexadentate. In contrast, *S. aureus* NCTC8325, the parental strain of a frequently manipulated strain RN4220, showed increased capacity for degradation of fatty acids. Therefore, in M49 strains, most activated acyl donors must be created by the fatty acid biosynthesis pathway. The analysis of these pathways revealed M49 to have all necessary enzymes for these pathways, which led us to investigate the contribution of glycolytic compounds on the BLI signal.

### Dependence of Luminescence on Glucose Homeostasis

**FIGURE 4:**
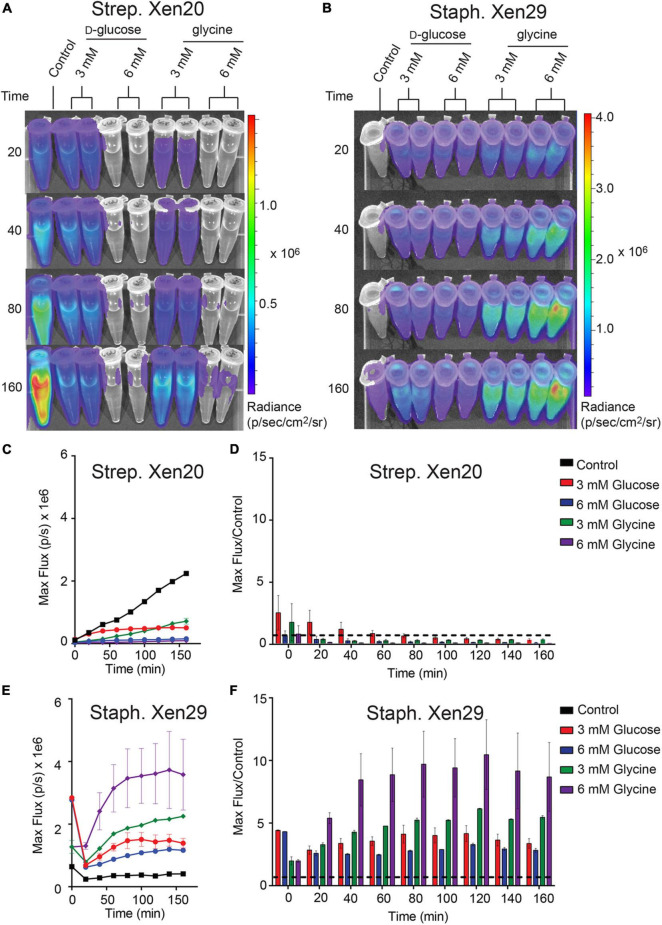
The effect of monosaccharides on bioluminescence production by Gram-positive pathogen. **(A)** Representative time course of *Strep*. Xen20 or *Staph.* Xen29. For all samples, cells were grown to the stationary phase then diluted in fresh phosphate-buffered saline (i.e., control) supplemented with either *D*-glucose or glycine as indicated. **(B)** Similar time course for *Staph*. Xen29. **(C)** Quantification of the *Strep*. Xen20 luminescent signal from **(A)**. **(C,D)** Quantification of luminescent signal from samples shown in panel **(A)** for *Strep*. Xen20 represented as either the maximum rate of photons generated (shown on the left as max flux) or as the relative maximum signal normalized to the control (shown on the right as flux/control) displayed as a function of time. The black dashed line indicates a ratio at the threshold of 1.0. **(E,F)** Similar quantification of panel **(B)** for *Staph*. Xen29. All samples are color coded as indicated and the experiment performed, as shown in the “Materials and Methods” section.

*Strep.* Xen20 processes sugars by homolactic fermentation, a process that utilizes *D*-glucose as the preferred carbon source (Levering et al., 2012). Based on KEGG genome available data, glycine is converted to pyruvate *via* conversion to serine by *L*-serine dehydratase in reactions essential to the formation of one-carbon metabolites. Therefore, we utilized *D*-glucose as a glycolytic carbon source and glycine as a non-glycolytic carbon source. *D*-glucose is present at physiological concentrations ranging between 3.9 and 5 mM in healthy adult mice (Lee and Bressler, 1981). Therefore, we tested exogenous *D*-glucose and glycine at either 3 or 6 mM (Figures 4A,B). The addition of either exogenous *D*-glucose and glycine to *Strep.* Xen20 decreased the BLI signal (Figures 4C,D). In contrary to this, the addition of *D*-glucose or glycine increased the luminescence expression in *Staph.* Xen29 (Figures 4E,F).

### Physiologically Relevant Limitations in Bioluminescence Can Be Attributed Solely to Metabolites Upstream of Pyruvate

**FIGURE 5:**
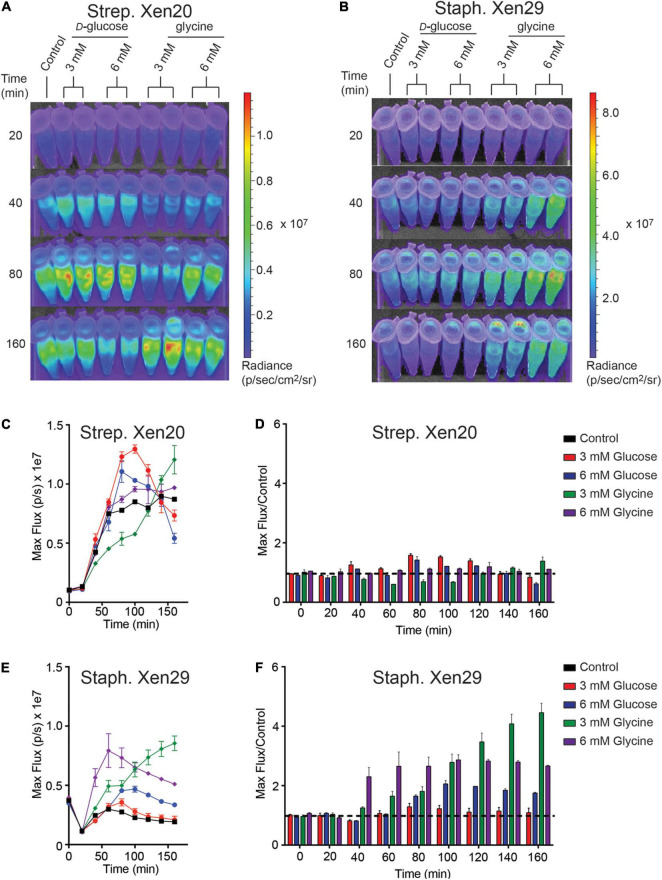
Increased luminescence production by Gram-positive pathogen incubated in M9 medium supplemented with casein hydrolysate and yeast extract. OD_600 *nm*_ and luminescence were measured using a Thermo Fischer VarioSkan plate at 20 min intervals over 160 min at a constant 37°C. **(A)** Representative time course of *Strep.* Xen20. For all samples, cells were grown to stationary phase then diluted in fresh M9 medium containing casein hydrolysate and yeast extract (i.e., control) supplemented with either *D*-glucose or glycine as indicated. **(B)** Similar time course for *Staph.* Xen29. **(C,D)** Quantification of the *Strep*. Xen20 luminescent signal from **(A)** represented as either the maximum rate of photons per second (max flux) and as the relative maximum signal normalized to the control (shown on the right as flux/control) displayed as a function of time. The black dashed line indicates a ratio at the threshold of 1.0. **(E,F)** Similar quantification of panel **(B)** for *Staph.* Xen29. All samples are color coded as indicated and the experiment performed, as shown in the “Materials and Methods” section.

To test tissue-derived carbon sources, we perturbed the carbon source equilibrium of both light-producing pathogens (*Strep.* Xen20 and *Staph.* Xen29) by characterizing the glucose dependency of BLI signal. The dose-dependent effect of *D*-glucose on light production was monitored following incubation with M9 minimal media supplemented only with casein hydrolysate and yeast extract (Figure 5). The supplements meant to provide the microbe with amino acids and cofactors essential for protein and DNA synthesis. In this moderately enhanced M9 medium, we found that *Strep.* Xen20 produced detectable levels of luminescence. Providing an additional glycolytic carbon source, in the form of *D*-glucose, did not significantly increase radiance. In contrast, *Staph.* Xen29 produced only moderate levels of luminescence in the absence of further carbon sources. The addition of 6 mM *D*-glucose significantly increased the production of light in *Staph.* Xen29. Similar to *D*-glucose, *Strep.* Xen20 grown in modified M9 alternatively supplemented with glycine (3 mM) produced no significant increase in BLI signal. In contrast, all supplementations increased bioluminescent expression in *Staph*. Xen29 cells with a maximal BLI signal when glycine was present (Figure 5).

### Restriction of Luminescence Is Dependent on *L-Lactate* Dehydrogenase

**FIGURE 6:**
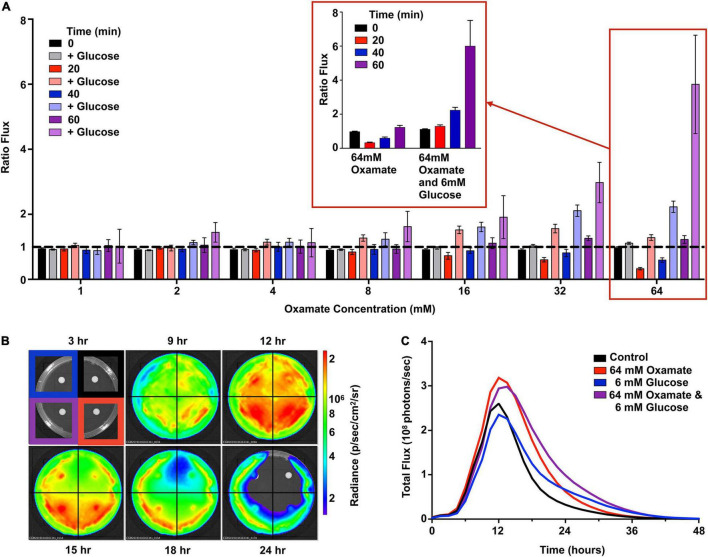
Oxamate inhibition partially recovers the bioluminescent signal. **(A)** *Strep*. Xen20 in the stationary phase were combined with PBS alone (i.e., control) or PBS with concentrations of 6 mM glucose and 0–64 mM oxamate. Represented is the flux from cells treated with oxamate divided by the flux from cells in PBS alone (ratio flux). The data represent seven trials without or without glucose at the indicated times. Expansion of the 64 mM oxamate concentration with and without glucose over 60 min shown (denoted in red inset). **(B)** *Strep*. Xen20 was grown on LB agar with disks containing either a water control (upper right), 64 mM oxamate (lower right), 6 mM glucose (upper left), or both glucose and oxamate (lower left) monitored for 48 h. **(C)** Quantification of the luminescent signal from panel **(B)** as the total rate of photons generates (total flux).

Homolactic fermentation produces pyruvate that is either converted to acetyl-CoA by pyruvate dehydrogenase or to lactate by *L*-lactate dehydrogenase (LDH, blue box, Figure 3). Pyruvate conversion by pyruvate dehydrogenase would prepare substrates relevant to light production. In contrast, conversion by LDH would scavenge pyruvate away from this potential pool. To test the effect of pyruvate conversion to lactate and the availability of the acetyl-CoA pool, various concentrations (0–64 mM) of oxamate, an inhibitor of LDH, were added to *Strep*. Xen20 cells (Figure 6; Feldman-Salit et al., 2013). However, luminescence was constant up to 32 mM oxamate (Figure 6A). At 64 mM oxamate, the radiance increased for the glucose-containing sample, but there was also an observed decrease in cell density. A diffusion assay tested the effect that drug eluted into agar has on light production (Figure 6B). Equal CFUs of *Strep.* Xen20 cells were added to the quadrants of an agar plate, and an oxamate or relevant control discs were added (Figure 6B). Discs were then imbued with equivalent volumes applied to them (20 μl), and the solutions were sterile water, glucose alone (0.022 mg), oxamate (0.14 mg), and both glucose and oxamate. Dried discs were placed on the agar plates. Bioluminescence production was higher in quadrants with oxamate (Figure 6C).

### Determining Plasma Glucose Concentrations Based on Relative Luminescence

**FIGURE 7:**
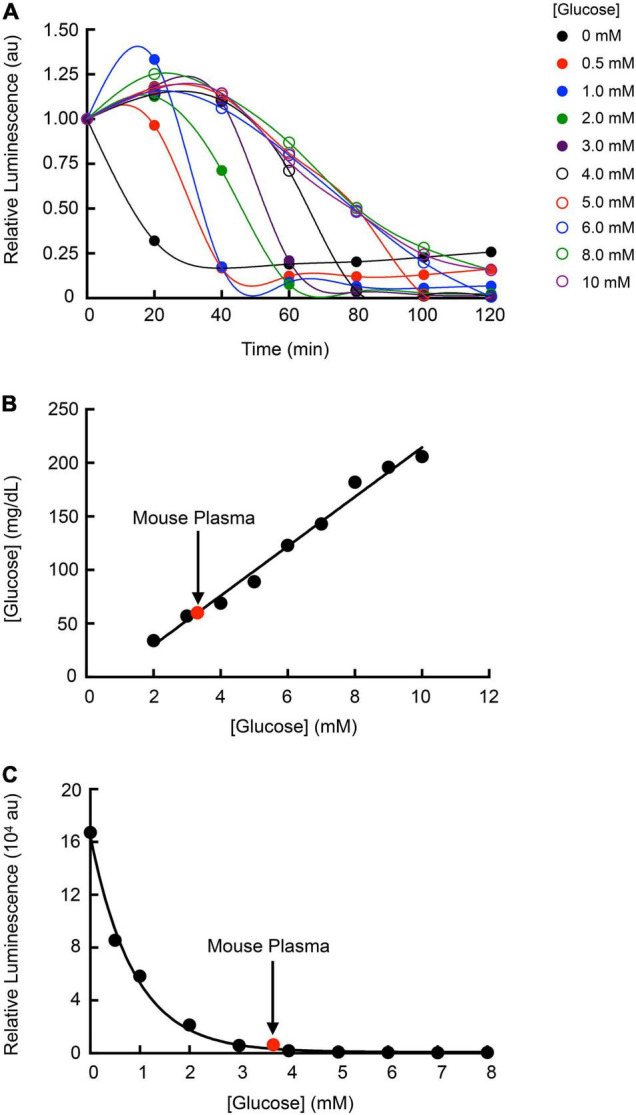
Utilizing *Strep.* Xen20 bioluminescence production to estimate plasma glucose concentrations. **(A)** *Strep.* Xen20 cells in the stationary phase were added to equal volumes of PBS with 0–10 mM glucose. The luminescence was measured with a luminometer every 20 min for 2 h. Curves through the points are connected *via* a cubic spline, and no parameters were obtained. **(B)** The glucose levels (mg/dl) of each PBS and glucose solution were measured using a commercially available blood glucose meter. Mouse plasma (indicated on graph in red) was also tested for blood glucose levels, which were used to estimate millimolar concentration. **(C)** Final luminescence in the *Strep.* Xen20 sample in glucose between 0 and 8 mM after incubation overnight. The exponential relationship yielded an equation of y = 16,7000e^×0.882x^ (*R*^2^ = 0.9861) and was used to estimate the mouse plasma glucose level based on luminescence of the *Strep.* Xen20.

Given the dependence of *Strep.* Xen20 bioluminescence on glucose homeostasis, we sought to correlate glucose concentration with the relative light production of *Strep.* Xen20. Light production of *Strep.* Xen20 decreased at relatively similar rates when added to an equal volume of glucose in phosphate-buffered saline (PBS). However, each sample displayed a different time to initial decrease and terminal luminescence (Figure 7A). Glucose concentration in the PBS and mouse plasma samples was confirmed using a commercial blood glucose meter. Based on the linear relationship, the mouse plasma had a glucose concentration of 3.3 mM (Figure 7B). After the *D*-glucose standards were allowed to incubate with the *Strep.* Xen20 culture overnight, an exponentially decreasing relationship was found to exist between the amount of glucose and *Strep.* Xen20 BLI (Figure 7C). The data were analyzed using a single exponential and used to predict glucose concentration in mouse plasma. Our predicted concentration of glucose in the mouse plasma matched actual glucometer values determined independently.

## Discussion

Competition for carbon sources and energy may affect the survival of *S. pyogenes* during an infection. Previously, we found that a *luxABCDE*-incorporated *S. pyogenes* had reduced light production in *D*-glucose-enriched medium (Davis et al., 2015). Although this trait is less desirable for *Strep.* Xen20 utility in non-invasive imaging of infections, it highlights a unique ability of bacterial luciferase to serve as a biosensor of activated or repressed metabolic pathways. We explored the use of *Strep.* Xen20 as a biosensor that could respond to changes in its local environment by altering its light production. This biosensor would fundamentally differ from the current utilization of isolated luciferases in biochemical assays. Specifically, our biosensor reports the activity of metabolic enzymes (substrates for light production) rather than on the level of promoter activity intrinsic to firefly or Renilla luciferase (under a bolus of the substrate).

Initially, genetic evidence pointed to *iolE* as the gene sequence interrupted by the *luxABCDE* pathway (Park et al., 2003). Results of inositol supplementation (Figure 2) indicate a genetic regulation of the *luxABCDE* bioluminescence. According to the KEGG metabolic pathways for NZ131, M49 serotype strains of *Strep.* Xen20 do not contain the enzymes necessary to convert this inositol further into usable carbon sources to enter glycolysis or the citric acid cycle. Therefore, we tested the effects of other carbon sources to link the creation of luminescent substrates to the central metabolism of the bacterial cell.

Our analysis of the KEGG metabolic pathways for NZ131 revealed no enzymes capable of fatty acid degradation beyond the creation of hexadecanoyl-CoA (see the scheme in Figure 3). Necessary activated fatty acyl compounds must then be created *via* the fatty acid biosynthesis pathway, which utilizes acetyl-CoA as building blocks for the creation of fatty acid chains. As previously mentioned, acetyl-CoA is produced in *S. pyogenes via* homolactic fermentation. Taken together, this suggests *S. pyogenes* has a more simplified route to the production of the essential light-producing building block than its counterpart *S. aureus*.

Glucose is utilized in one of the two ways, namely, first, it can be transported into the cell by phosphotransferases, which convert it to glucose-6-phosphate during uptake, and fed into the fermentation process; second, it can be converted to glucose-6-phosphate by hexokinase (Levering et al., 2012). The addition of *D*-glucose and glycine was shown in this study to decrease the bioluminescence of *Strep.* Xen20. As seen in Figure 3 and reviewed elsewhere (Levering et al., 2012), excess glucose during homolactic fermentation causes the conversion of pyruvate to lactate by the enzyme LDH due to increased levels of fructose-bisphosphate (FBP), intensified by the inhibition of pyruvate conversion to acetyl-CoA by increased triose phosphate levels. Therefore, the decreased bioluminescence observed in *D*-glucose supplementation may be due to the reduced acetyl-CoA pool and subsequent lack of fatty acid biosynthesis precursors. As such, we tested the addition of an inhibitor of LDH to attempt to restore the acetyl-CoA pool and improve the luminescent signal (Feldman-Salit et al., 2013). Oxamate-containing discs allowed for the increased light production in the dispersion area (Figure 6). Therefore, bioluminescence is diminished in the presence of increased glucose due at least in part to its generation of FBP. This FBP acts on LDH to scavenge the pool of available pyruvate, committing it to the production of lactate rather than the fatty acyl precursor acetyl-CoA. Glycine supplementation showed comparable bioluminescence levels at concentrations equal to those of *D*-glucose. Although glycine is first converted to pyruvate, gluconeogenesis can convert this pyruvate back to triose phosphate and/or FBP, creating a similar effect to *D*-glucose supplementation.

Interestingly, the addition of amino acid sources, such as casein hydrolysate, relaxed the inhibition of bioluminescent signal by *D*-glucose and glycine supplementation (Figure 5). According to the KEGG diagram for *S. pyogenes* NZ131, *L*-alanine can be converted to pyruvate and can contribute to luminescence. In contrast, amino acids that are converted to fumarate are unable to contribute due to the lack of citrate cycle activity. Leucine, valine, and isoleucine are converted to their subsequent oxopentanoates, but subsequent reactions are not possible due to a lack of 3-methyl-2-oxobutanoate dehydrogenase. It is unclear which amino acid or a combination of amino acids plays a role in increasing the *Strep.* Xen20 light production in the presence of glucose and, as such, dissection of that would require further study.

The application of the data gathered in this study led us to investigate how *Strep.* Xen20 might be used as a living sensor of local glucose concentration. The results of our glucose standards and mouse plasma glucose levels give proof-of-concept evidence that *Strep.* Xen20 can be used to determine the approximate levels of blood glucose in a sample, similar to the heme sensing capabilities of the luminescent *E. coli* (Mimee et al., 2018). In this study, we evaluated the *Strep.* Xen20 luminescence using mouse plasma. However, it is entirely feasible that *Strep.* Xen20 could detect glucose in other species and in the host. Additionally, incorporating the *lux* operon in other homofermentative bacteria such as *Lactococcus lactis or Streptococcus thermophilus* could prove useful for additional research or as other options for biosensor candidates.

To the best of our knowledge, these results are the first to highlight the effects of the central metabolism on the bacterial luciferase system. It is conceivable that directed insertion of the *luxABCDE* operon could generate a biosensor that reporters on the presence of trace heavy metals or the presence of toxicity compounds, such as arsenic (De Mora et al., 2011; Huang et al., 2015). It would be easy to ignore the complications of light production by using non-integrated plasmid versions of LuxAB in these pathogens and injection luciferin substrate, such as the *ffluc* system previously described in *S. pyogenes* (Loh and Proft, 2013). This would be simpler, in many regards, but future correlations of metabolism-dependent light production with RNA-seq technology could provide new avenues for the high-throughput assessment of compounds that disrupt molecular pathways regulating these dangerous pathogens.

## Materials and Methods

### Chemicals and Reagents

Brain heart infusion (BHI), sheep blood agar (SBA), and THY were from BD Biosciences (San Jose, CA) or RPI. Kanamycin and *D*-glucose were purchased from Research Products International (RPI; Mt. Prospect, IL). Glycine was purchased from AMERSCO (Solon, OH). Unless otherwise specified, all reagents were purchased from Sigma Aldrich. M9 minimal medium was prepared as previously described (Miller, 2010).

### Bacterial Strains, Cultivation Conditions, and Imaging

Strains *Strep.* Xen20 and *Staph.* Xen29 (PerkinElmer Inc., Waltham, MA) were grown to stationary phase (OD_600*nm*_ > 1) in BHI broth for 18 h at 37°C, and approximate concentration was determined by light scattering at OD_600 *nm*_ per manufacturer’s instructions. Cultivation conditions were altered either in the liquid media or on the solid media and supplemented with additives, as indicated. Plates and tubes were imaged using the IVIS Lumina XRMS or Lumina II system (PerkinElmer Inc.). BLI was reported as calibrated units of radiance (p/sec/cm^2^/sr) using the LivingImage software version 4.7 (PerkinElmer Inc.), allowing for comparisons between detectors.

### Bioluminescence Kinetic Assays

To test if the *lux* operon was, in fact, under *iolE* (Park et al., 2003), we diluted bacteria in PBS containing myo-inositol, *D*-glucose, or both in a final 1 ml volume at equivalent OD_600 *nm.*_ Tubes were incubated and imaged in the IVIS Lumina XRMS on a 37°C stage and imaged every 20 min for 160 min. *Strep*. Xen20 was also streaked on a secondary set of plates with LB (RPI) alone or supplemented with either 6 mM myo-inositol, 50 mM *D*-glucose, or both myo-inositol and *D*-glucose. Plates were incubated at 37°C and imaged at 14, 24, and 38 h. Catabolite repression was assessed by supplementation of agar with myo-inositol or *D*-glucose or both in 6-well, flat-bottomed culture plates (Costar, Corning, NY) and imaged every 1.5 h with the IVIS Lumina II for 48 h.

The effect of carbon source availability was determined by incubating cells in sterile PBS (i.e., control) or PBS supplemented with either 3 mM or 6 mM of *D*-glucose or glycine. Sample tubes were diluted and imaged as before. To determine the contribution of growth rate to *Strep*. Xen20 expression, this process was repeated for M9 minimal medium supplemented with 1% casein hydrolysate and 0.3% yeast extract. This media provides necessary cofactors for growth in the absence of confounding carbon sources.

### Oxamate Inhibition Assay

To determine the contribution of LDH on *Strep.* Xen20 bioluminescence, *Strep*. Xen20 cells in the stationary phase were added to PBS containing a final concentration of 6 mM *D*-glucose in a 96-well plate. Varying amounts of oxamate were added for final concentrations of 0–256 mM. OD_600 *nm*_ and luminescence were measured using a Thermo Fischer VarioSkan plate at 20 min intervals over 160 min at a constant 37°C. For comparison purposes, cells treated with oxamate were compared with those untreated in both the control and glucose-supplemented groups. In addition, *Strep.* Xen20 was grown and imaged for bioluminescence over 48 h on an LB agar plate containing discs saturated with solutions containing 6 mM oxamate, 6 mM *D*-glucose, or both. The discs were prepared by the addition of 4 equal volume solutions (20 μl) corresponding to sterile water, glucose alone (0.022 mg), oxamate (0.14 mg), and both glucose and oxamate. The discs were dried before placing them on the agar plates.

### Plasma Glucose Effect on Bioluminescence

To evaluate *Strep.* Xen20 as a potential monitor of local plasma glucose concentrations, *Strep.* Xen20 grown to the stationary phase in BHI was combined in a 1:1 ratio with glucose in PBS at concentrations between 0 and 10 mM. *Strep.* Xen20 was also combined with C57BL6 mouse plasma (Innovative Research, Novi, MI). The light production was then monitored at 20 min intervals at 37°C for 2 h using the Glomax 20/20 Luminometer (Promega, Madison, WI). A final measurement was also taken after the samples had been allowed to incubate overnight at room temperature. The glucose level in the glucose standards and mouse plasma were also measured directly using a commercially available ReliOn™ PRIME blood glucose meter (Wal-Mart Stores, Inc., Bentonville, AR) ReliOn PRIME blood glucose test strips. The meter was unable to determine the glucose levels at < 2 mM.

### Statistical Analysis

Statistical means of each group were analyzed using two-way, repeated-measures analysis of variance (ANOVA) on R 3.1.2.^2^

Time, substrate, and the interaction of time × substrate were analyzed for each medium and each bacterium. Results were reported for statistical values *p* < 0.05 or no significant difference for *p* > 0.05.

## Data Availability Statement

The raw data supporting the conclusions of this article will be made available by the authors, without undue reservation.

## Author Contributions

RD, CM, HE, MH, and PP designed and conducted the experiments. RD, CM, and PP analyzed the data. All authors contributed to the writing and editing of this manuscript.

## Conflict of Interest

The authors declare that the research was conducted in the absence of any commercial or financial relationships that could be construed as a potential conflict of interest.

## Publisher’s Note

All claims expressed in this article are solely those of the authors and do not necessarily represent those of their affiliated organizations, or those of the publisher, the editors and the reviewers. Any product that may be evaluated in this article, or claim that may be made by its manufacturer, is not guaranteed or endorsed by the publisher.

## Footnotes

1
http://www.genome.jp/dbget-bin/www_bget?genome:T00780

2
http://www.r-statistics.com/on/r/

## Abbreviations

*S. pyogenes*: *Streptococcus pyogenes*
*luxABCDE*: *Photorhabdus luminescens lux* operon cassette
*ffluc*: firefly luciferase
*Strep*. Xen20: *Streptococcus pyogenes* Xen20
*E. coli*: *Escherichia coli*
FMNH_2_: reduced flavin mononucleotide
THY: Todd-Hewitt with yeast extract
SB: sheep blood
GFP: green fluorescent protein
KEGG: Kypto Encyclopedia of Genes and Genomes
CoA: coenzyme A
BHI: brain-heart infusion
SBA: sheep blood agar
ANOVA: analysis of variance
LB: Luria broth
PBS: phosphate buffer saline
NAD^+^: nicotinamide adenine dinucleotide
triose-P: triose phosphate
HPr: histidine-containing carrier protein
HPr: HPr(Ser-P)
PEP: phosphoenolpyruvate
BPG: 1,3-bisphosphoglyceric acid.
